# GSK3α Regulates Temporally Dynamic Changes in Ribosomal Proteins upon Amino Acid Starvation in Cancer Cells

**DOI:** 10.3390/ijms241713260

**Published:** 2023-08-26

**Authors:** Lorent Loxha, Nurul Khalida Ibrahim, Anna Sophie Stasche, Büsra Cinar, Tim Dolgner, Julia Niessen, Sabine Schreek, Beate Fehlhaber, Michael Forster, Martin Stanulla, Laura Hinze

**Affiliations:** 1 Department of Pediatric Hematology and Oncology, Hannover Medical School, 30625 Hannover, Germany; loxha.lorent@mh-hannover.de (L.L.); ibrahim.nurul@mh-hannover.de (N.K.I.); stasche.anna@mh-hannover.de (A.S.S.); cinar.buesra@mh-hannover.de (B.C.); dolgner.tim@mh-hannover.de (T.D.); niessen.julia@mh-hannover.de (J.N.); schreek.sabine@mh-hannover.de (S.S.); fehlhaber.beate@mh-hannover.de (B.F.); stanulla.martin@mh-hannover.de (M.S.); 2 Institute of Clinical Molecular Biology, Kiel University, 24105 Kiel, Germany; m.forster@ikmb.uni-kiel.de

**Keywords:** GSK3α, Wnt/STOP, asparaginase, amino acid starvation, metabolism, cancer, acute leukemia, colorectal cancer, ribosomal proteins, gene regulation

## Abstract

Amino acid availability is crucial for cancer cells’ survivability. Leukemia and colorectal cancer cells have been shown to resist asparagine depletion by utilizing GSK3-dependent proteasomal degradation, termed the Wnt-dependent stabilization of proteins (Wnt/STOP), to replenish their amino acid pool. The inhibition of GSK3α halts the sourcing of amino acids, which subsequently leads to cancer cell vulnerability toward asparaginase therapy. However, resistance toward GSK3α-mediated protein breakdown can occur, whose underlying mechanism is poorly understood. Here, we set out to define the mechanisms driving dependence toward this degradation machinery upon asparagine starvation in cancer cells. We show the independence of known stress response pathways including the integrated stress response mediated with GCN2. Additionally, we demonstrate the independence of changes in cell cycle progression and expression levels of the asparagine-synthesizing enzyme ASNS. Instead, RNA sequencing revealed that GSK3α inhibition and asparagine starvation leads to the temporally dynamic downregulation of distinct ribosomal proteins, which have been shown to display anti-proliferative functions. Using a CRISPR/Cas9 viability screen, we demonstrate that the downregulation of these specific ribosomal proteins can rescue cell death upon GSK3α inhibition and asparagine starvation. Thus, our findings suggest the vital role of the previously unrecognized regulation of ribosomal proteins in bridging GSK3α activity and tolerance of asparagine starvation.

## 1. Introduction

Cancer cells inevitably encounter stress due to excessive proliferation rates, which raise the demand for nutrient availability and protein synthesis. Some cancers, such as acute lymphoblastic leukemia (ALL), depend on asparagine availability to maintain cell survival, which is exploited clinically with the use of the bacterially derived enzyme asparaginase that depletes asparagine [1,2,3,4].

However, tolerance of amino acid depletion can cause cancer cell resistance and thus represents a major clinical obstacle. An in-depth characterization of cellular signaling pathways is essential to understand the regulatory mechanisms of cellular homeostasis in response to amino acid deprivation.

In previous studies, we could demonstrate that resistant leukemia cells, as well as colorectal cancer cells (CRC), rely on GSK3-dependent protein degradation as an alternative source of amino acids to maintain cellular fitness upon amino acid depletion. The inhibition of GSK3-dependent protein degradation leads to the activation of a non-canonical branch of Wnt signaling, termed Wnt-dependent stabilization of proteins (Wnt/STOP) [5], that mediates cell death in the presence of amino acid scarcity [6,7,8]. Importantly, we found that asparaginase sensitization is solely dependent on the alpha isoform of GSK3 [6,7,8]. Due to its role in different cancer entities, GSK3α thus has a pivotal role in regulating the cellular response to amino acid deprivation. However, cancer cells can develop tolerance toward GSK3α inhibition and asparagine depletion, whose mechanistic underpinnings are not sufficiently understood. Thus, we set out to define molecular factors that drive or inhibit cell death upon GSK3α inhibition and asparagine starvation in cancer cells.

Environmental stressors, such as amino acid shortage, are well known to cause the accumulation of misfolded or unfolded proteins, resulting in endoplasmic reticulum (ER) stress [9,10]. The unfolded protein response (UPR) is a cellular adaptive response that evolved to restore protein-folding homeostasis by reducing protein synthesis and by increasing ER protein folding [11]. Protein ubiquitination and proteasomal degradation are important for the degradation of unfolded or damaged proteins [12]. However, despite its link to proteasomal degradation, the activation of Wnt/STOP and asparaginase treatment has been shown to not affect established UPR markers such as XBP1 mRNA splicing and PERK phosphorylation [10], arguing against activation of the UPR response as a mediator of cell death [7].

One central signaling node that controls the cellular response to amino acid availability is the evolutionarily conserved kinase GCN2 [13,14]. The key characteristic of GCN2 within the integrated stress response (ISR), a homeostatic system by which eukaryotic cells sense and respond to stress-inducing signals, is its role as a sensor of amino acid depletion [14,15,16]. Stress is then ameliorated by affecting changes in both global protein synthesis and the expression of certain key genes to either restore homeostasis or induce apoptosis [11,17]. Depending on the length and severity of stress, the response can be directly pro-survival, activating genes that oppose the infringing stress and promote a return to homeostasis, or instead can induce apoptosis if survival is not possible [17,18,19,20]. While the activation of GCN2 upon starvation has been shown to inhibit global protein translation, some selected transcripts, such as the cellular transcriptional factor ATF4, can display an increase in translation [21]. Thus, in order to respond to amino acid depletion effectively, amino-acid-synthesizing enzymes, such as the asparagine synthesizing enzyme ASNS, and transporter genes are under the control of the GCN2-ATF4 pathway [22,23]. The GCN2-ATF4 pathway is critical for tumor cell survival and proliferation when challenged by acute amino acid deprivation [18,24]. While the acute response has been extensively studied, the activation in the presence of chronic stress is less defined. Upon chronic starvation, the ATF4 axis has been demonstrated to be pro-apoptotic through the upregulation of CHOP [25]. Thus, we explored whether cell death mediated by GSK3α inhibition and asparaginase treatment is dependent on the GCN2 axes. However, we found no upregulation of ASNS nor dependence on GCN2 or CHOP activity, indicating independence from the acute and chronic GCN2-ATF4 branches.

Instead, we show that GSK3α inhibition leads to the temporally dynamic downregulation of ribosomal proteins upon amino acid starvation. Ribosome biogenesis is a highly coordinated process involving the synthesis and processing of ribosomal RNA (rRNA), the synthesis of ribosomal proteins and their import into the nucleus, the assembly of ribosome subunits, and the transport of the mature 40S (composed of RPS) and 60S (composed of RPL) subunits into the cytoplasm [26,27,28]. In addition to their structural and regulatory roles in the translation machinery, RPs can perform other “moonlighting” extra-ribosomal functions including the regulation of cell growth, proliferation, and differentiation [29]. These functions are defined based on specific interactions between RPs with non-ribosomal cellular components independent of the ribosome [30,31,32,33,34]. In the context of extra-ribosomal functions, previous studies could demonstrate an intriguing pattern of RP expression in cancers. While several RP genes displayed pro-oncogenic effects and resulted in increased proliferation, other RP genes consistently exhibited negative dysregulation across cancers, which thereby acted directly or indirectly as tumor suppressors.

In line, we found that inhibition of distinct ribosomal proteins of the small and large subunits, which are known to display an anti-proliferative effect, could rescue GSK3-inhibited cells from asparaginase-induced cytotoxicity.

Thus, we can demonstrate a previously unrecognized link between ribosomal proteins and GSK3α activity in regulating the cellular response to amino acid starvation.

## 2. Results

### 2.1. Loss of GSK3α Induces Asparaginase Cytotoxicity Independent from ASNS Expression in Resistant Cancer Cells

**Figure 1:**
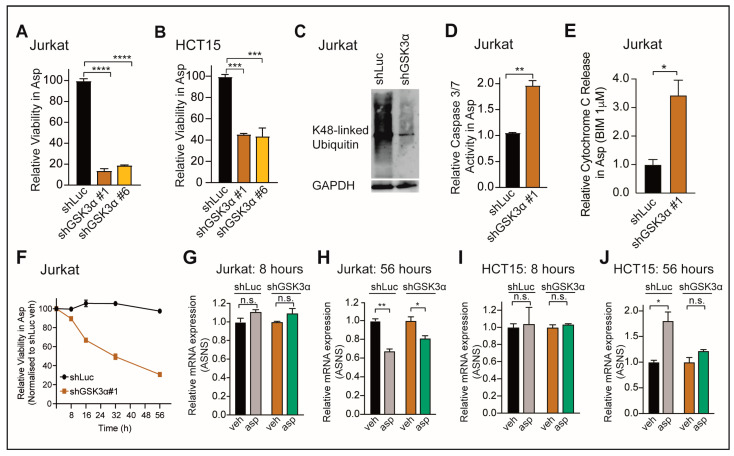
Loss of GSK3α induces asparaginase cytotoxicity independent from ASNS expression in resistant cancer cells. (**A**,**B**) Cells were transduced with indicated constructs and treated with vehicle or 100 U/L of asparaginase in biological triplicates. Relative viability was assessed after 8 days of treatment by counting viable cells. Note that an earlier time point was chosen as in [6] to allow for direct comparison between the two cell lines of different cancer entities. Statistical significance was assessed using a one-way ANOVA with Dunnett’s adjustment for multiple comparisons. (**C**) Jurkat cells were transduced with indicated shRNAs. Upon knockdown validation, protein levels of K48-linked ubiquitin and GAPDH were assessed using Western blot analysis. (**D**) Indicated cells were transduced with indicated shRNAs, treated with vehicle or 100 U/L of asparaginase for 48 h, and Caspase 3/7 activity was assessed in biological triplicates. Statistical significance was assessed using a two-sided Student’s *t*-test with Welch adjustment. (**E**) Cells were transduced with indicated shRNAs and treated with 100 U/L of asparaginase for 48 h, and cytochrome C release was assessed in biological triplicates. Statistical significance was assessed using a two-sided Student’s *t*-test with Welch adjustment. (**F**) Jurkat cells were transduced with indicated shRNAs and treated with vehicle or 100 U/L of asparaginase in biological triplicates. Relative viability was assessed at indicated time points by counting viable cells. All cell counts were normalized to shLuc-transduced, vehicle-treated cells. (**G**–**J**) Cell lines were transduced with indicated shRNAs and treated with vehicle or 100 U/L of asparaginase at each indicated time point. Relative ASNS expression was assessed with qRT-PCR analysis in biological duplicates and normalized to each vehicle condition. Statistical significance was assessed using a two-sided Student’s *t*-test with Welch adjustment. **** *p* ≤ 0.0001, *** *p* ≤ 0.001, ** *p* ≤ 0.01, * *p* < 0.05, and n.s. *p* ≥ 0.05.

To define factors that drive dependence toward the GSK3α-dependent proteasomal degradation machinery, we started by inducing a knockdown of GSK3α in Jurkat T-ALL cells as well as in the colorectal cancer (CRC) cell line HCT15. The knockdown of GSK3α using two independent shRNAs in Jurkat (Figure S1A), as well as in HCT15 cells (Figure S1B), resulted in a striking asparaginase sensitization (Figure 1A,B), which could be rescued by expressing the GSK3α wild-type (WT) sequence (Figure S1C,D), indicating an on-target effect [7]. The effect of the knockdown was also evident with the decrease in K48-linked ubiquitin levels (Figure 1C and Figure S1E), which is one of the well-established hallmarks of an activated Wnt/STOP pathway [5,6,7,35]. In both cell lines, the inhibition of GSK3α and asparaginase treatment displayed a robust increase in mitochondrial apoptosis, as assessed with Caspase 3/7 activity (Figure 1D) or BH3 profiling (Figure 1E and Figure S1F).

Expression levels of the asparagine-synthesizing enzyme, ASNS, have long been attributed to sensitivity and resistance to asparaginase. However, studies could demonstrate that ASNS expression and asparaginase response are poorly correlated in human leukemia cells [36,37,38,39]. Interestingly, ASNS expression has been shown to rapidly increase through activation of the transcription factor ATF4 as an acute and immediate cellular response to amino acid deprivation [22,23]. We thus asked whether cell death in the context of asparagine scarcity and loss of GSK3α involves changes in the expression levels of ASNS.

To address this question, we treated Jurkat, as well as HCT15 cells, with asparaginase in the presence or absence of GSK3α inhibition and subsequently assessed ASNS mRNA expression levels. Given the fact that amino acid deprivation has to be present for several days to observe the above-described sensitization phenotype, we assessed ASNS expression levels not only at an early time point but also after 56 h, at which we were able to observe at least 50% cell death (Figure 1F and Figure S1G). However, we failed to observe any significant differences in GSK3α-inhibited cells upon asparagine depletion (Figure 1G–J). Thus, these findings collectively argue against the role of ASNS expression in mediating GSK3α-dependent asparaginase cytotoxicity.

### 2.2. GSK3α-Mediated Response to Chronic Amino Acid Deprivation Is Independent of the GCN2-CHOP Axis

Next, we asked whether GSK3α inhibition mediates cancer cell death in response to asparagine depletion through direct pro-apoptotic signaling. Previous studies could demonstrate that uncharged tRNAs, which accumulate intracellularly during amino acid limitation, activate the protein kinase GCN2, a well-known regulator of translation in amino-acid-starved cells that phosphorylates the eukaryotic initiation factor 2α (eIF2α) [14,40]. eIF2α phosphorylation can inhibit global protein translation and induce the translation of specific transcripts such as ATF4 [21]. ATF4 can then function to stimulate the expression of target genes [22,23] to increase amino acid synthesis and protein folding. In the context of acute activation, GCN2 serves as a pro-survival signal [18,40,41], whilst the effect of a chronic GCN2 activation remains ill-defined.

**Figure 2:**
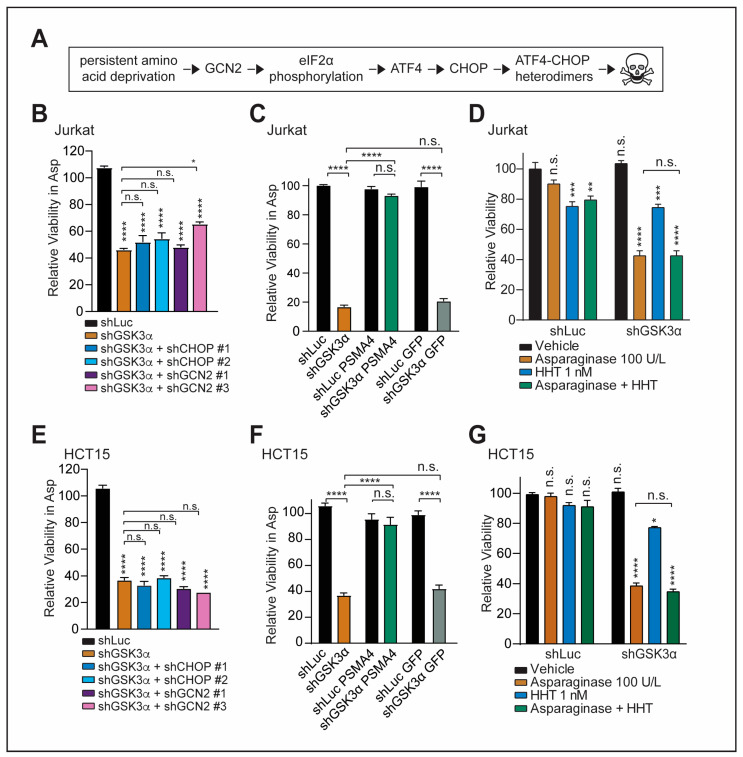
GSK3α-mediated response to chronic amino acid deprivation is independent of the GCN2-CHOP axis. (**A**) Schematic depiction of the GCN2-CHOP axis in the context of chronic amino acid deprivation. (**B**) Jurkat cells were transduced with indicated shRNAs and treated with vehicle or 100 U/L of asparaginase in biological triplicates. Relative viability was assessed after 6 days of treatment by counting viable cells. All cell counts were normalized to vehicle-treated cells. Treatment was concluded at an earlier time point due to the toxicity of GCN2 and CHOP knockdown at a later time point. (**C**) Jurkat cells were transduced with indicated constructs and treated with vehicle or 100 U/L of asparaginase. Relative viability was assessed after 8 days of treatment by counting viable cells. All cell counts were normalized to shLuc-transduced, vehicle-treated cells. (**D**) Jurkat cells were transduced with indicated constructs and treated with indicated treatments in biological triplicates. Relative viability was assessed after 6 days of treatment by counting viable cells. Counts were normalized to vehicle-treated cells. (**E**) HCT15 cells were treated as in (**B**). (**F**) HCT15 cells were treated as in (**C**). (**G**) HCT15 cells were treated as in (**D**). Statistical significance was assessed using a one-way ANOVA with Dunnett’s adjustment for multiple comparisons. **** *p* ≤ 0.0001, *** *p* ≤ 0.001, ** *p* ≤ 0.01, * *p* < 0.05, and n.s. *p* ≥ 0.05.

However, in our context, the chronic axis is a pertinent aspect to be addressed as the induction of cell death upon GSK3α inhibition in cancer cells involves persistent asparagine depletion. Prolonged starvation has been shown to induce apoptosis through the activation of ATF4 (Figure 2A) [11,20]. This leads to subsequent upregulation of the pro-apoptotic transcription factor CHOP with a resulting formation of ATF4-CHOP heterodimers that can (i) activate further downstream pro-apoptotic targets and (ii) drive protein translation leading to ATP depletion and cell death [11,25,42]. For instance, glutamine starvation in MYC-mediated neuroblastoma has been shown to induce apoptosis through the GCN2-ATF4 branch [43].

Thus, we first wondered whether cell death in response to the loss of GSK3α and amino acid scarcity is mediated through a GCN2-CHOP-dependent axis. To test this experimentally, we started by inducing a knockdown of two downstream effectors of the axis, GCN2 and CHOP, in Jurkat T-ALL cells (Figure S1H). CHOP serves as the major pro-apoptotic effector of ATF4 activation in the ER stress response [44,45]. We thus reasoned that if cell death is mediated through this axis, a knockdown of the key effectors should block asparaginase sensitization induced by the inhibition of GSK3α. However, the knockdown of CHOP or GCN2 failed to rescue shGSK3α cells from asparaginase-induced cell death (Figure 2B). By contrast, expression of the hyperactive proteasomal subunit ∆N-PSMA4, which directly stimulates proteasomal degradation of a range of proteasomal substrates [46], served as a positive control [6,7] and was able to rescue shGSK3α cells from asparaginase cytotoxicity (Figure 2C). Importantly, all described findings could be independently validated in the colorectal cancer cell line HCT15 (Figure 2E,F and Figure S1I).

Second, to further strengthen the argument that GSK3α-mediated asparaginase response is independent of the ATF4-CHOP branch, we asked whether inhibition of protein synthesis can protect cells from the toxicity of GSK3α inhibition and asparagine depletion. This is due to the fact that ATF4-CHOP heterodimers can drive protein translation leading to ATP depletion and cell death [25]. However, treatment with the elongation inhibitor homoharringtonine failed to rescue cells from GSK3α-mediated cell death upon asparagine depletion in Jurkat T-ALL cells (Figure 2D) as well as in colorectal cancer cells (Figure 2G). Collectively, these data indicate that GSK3α inhibition mediates asparaginase sensitization independent of the GCN2-CHOP axis.

### 2.3. Cell Death upon Inhibition of GSK3α Is Not Mediated by Changes in Cell Cycle

The Wnt/STOP pathway is best known to regulate cell size and growth owing to its role in stabilizing proteins during mitosis. A previous study has shown that proteins were periodically stabilized at the G2/M cell cycle phase when Wnt/STOP was active [5]. This is essential for optimal cell cycle progression as cells require a sufficient amount of proteins in preparation for cell division.

**Figure 3:**
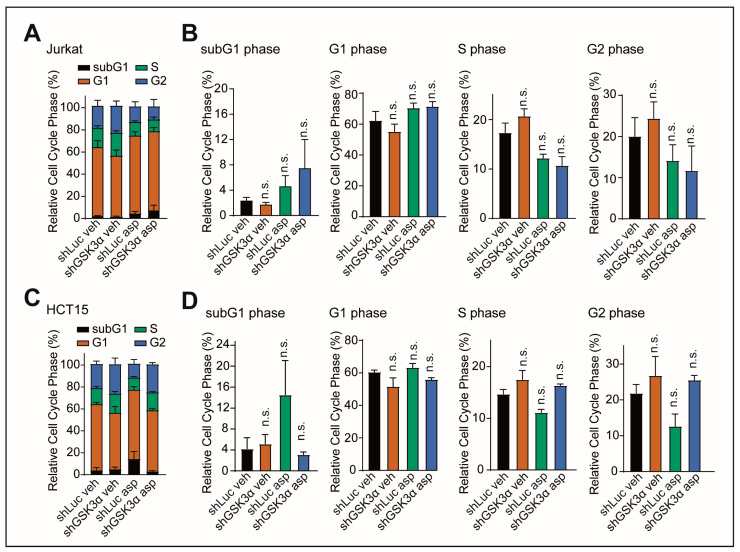
GSK3α inhibition and asparagine depletion do not cause changes in the cell cycle. (**A**) Jurkat cells were transduced with indicated shRNAs and treated with vehicle or 100 U/L of asparaginase. Cell cycle analysis was conducted after 48 h of treatment using flow cytometry in biological duplicates. (**B**) Statistical analysis of the cell cycle analysis from (**A**) for each cell cycle phase. Statistical significance was assessed using a one-way ANOVA with Dunnett’s adjustment for multiple comparisons. (**C**) HCT15 cells were treated and assessed as in (**A**). (**D**) Statistical analysis of the cell cycle analysis from (**C**) for each cell cycle phase assessed as in (**B**). n.s. *p* ≥ 0.05.

Thus, we asked whether distinct changes in the cell cycle could prompt the progressive direction of cell death. To test this, we determined cell cycle stages in Jurkat and HCT15 cells transduced with GSK3α shRNA. However, we did not find any significant effects in either the presence or absence of asparagine starvation (Figure 3A–D). This indicates that cell cycle changes do not influence the course toward cell death.

### 2.4. Inhibition of GSK3α Leads to Temporally Dynamic Downregulation of Distinct Ribosomal Proteins in the Presence of Asparagine Deprivation

**Figure 4:**
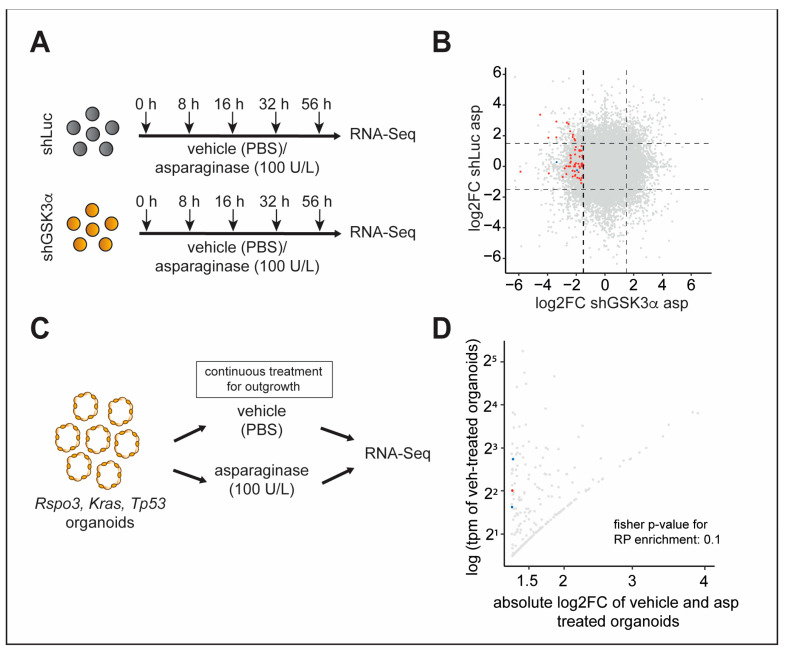
Inhibition of GSK3α leads to temporally dynamic downregulation of distinct ribosomal proteins in the presence of asparagine deprivation. (**A**) Schematic depiction of the workflow to obtain samples for RNA sequencing in Jurkat T-ALL cells. Cells transduced with indicated shRNAs were treated with vehicle or 100 U/L of asparaginase and sampled at each time point indicated. (**B**) Scatterplot showing the log2FC of comparisons between the untreated 0 h time point and treatment for all time points. A log2FC cut-off of 1.5 was used to identify differentially expressed transcripts, indicated with dashed lines. Red dots show RP transcripts that are differentially downregulated in shGSK3α cells while not being downregulated in shLuc cells. Blue dots indicate RP transcripts that were studied further. (**C**) Rspo3; Kras; and Trp53 mouse intestinal organoids were treated continuously with vehicle or asparaginase for 14 days. Upon outgrowth of organoids in the asparaginase-treated conditions, organoids were harvested and analyzed with RNA sequencing. (**D**) Scatterplot showing differentially downregulated transcripts in organoids from (**C**). Red dots indicate RP transcripts. Blue dots indicate RP transcripts that were independently validated in the CRISPR/Cas9 screen (Figure 5).

Next, we aimed to identify genes and biological processes regulated upon inhibition of GSK3α. In an exploratory approach, we first induced a robust GSK3α knockdown in Jurkat T-ALL cells and subsequently treated these cells with vehicle or asparaginase. To identify early, intermediate, and late responses to GSK3α inhibition in the presence or absence of asparagine depletion, we harvested cells at 8, 16, 32, and 56 h of treatment (Figure 4A) and performed gene expression analysis with RNA-sequencing. We chose these time points due to the gradual decrease in cell viability (Figure 1F and Figure S1G). This allowed the detection of early changes due to induction of cell death as well as changes at later time points with a small subset of remaining surviving cells.

Analysis of the RNA-sequencing results revealed that the expression of 2046 transcripts changed as early as 8 h after the start of asparaginase treatment. After 56 h of treatment, 1161 and 880 transcripts were differentially upregulated or downregulated, respectively (fold change > 1.5). Interestingly, the absolute number of differentially expressed transcripts did not grow significantly over time, while the constellation of transcripts that were differentially expressed changed between time points (Figure S2A).

Upon further analysis of differentially expressed transcripts, we observed that ribosomal proteins of the small (RPS) and large (RPL) subunits were significantly enriched (fisher *p* 7.44 × 10^−10^ at 56 h asparaginase treatment) in downregulated transcripts in the presence of an asparagine depletion in shGSK3α cells when compared to shLuc cells (fold change < −1.5 in shGSK3α while fold change > −1.5 in shLuc) (Figure 4B, Table S1).

Intriguingly, besides their role in the assembly of ribosomal components, ribosomal proteins can also perform other extra-ribosomal functions, including the regulation of cell proliferation [29,30,31,32,33,34]. These functions are defined based on specific interactions between RPs with non-ribosomal cellular components independent of the ribosome [30,31,32,33,34]. As described above, we observed a GCN2-CHOP independent phenotype and no effect with the elongation inhibitor homoharringtonine, indicating that driving protein translation with subsequent ATP depletion is unlikely to cause cell death in GSK3α-inhibited cells. Thus, the extra-ribosomal functions of RPs appeared to be an interesting axis for further investigation.

In the context of extra-ribosomal functions, previous studies could demonstrate an intriguing pattern of RP expression in cancers. While some RP genes display pro-oncogenic effects, other RP genes can act directly or indirectly as tumor suppressors [47]. For instance, some RP gene knockouts have been positively selected in a CRISPR-based viability screen carried out in a melanoma cancer cell line [48], indicating that RP gene loss is not always detrimental to cellular fitness. Thus, the loss of individual ribosomal proteins correlates with, and in some cases induces, specific effects on cellular proliferation.

Thus, we wondered whether the downregulation of RPS/RPL transcripts reflects a mechanism in cells that can survive amino-acid-deprived conditions in GSK3α-inhibited cells.

To address this question, we turned to the Ptprk–Rspo3 fusion of CRC organoids, which potentiate Wnt ligand-induced inhibition of GSK3. These cells are known to be highly asparaginase sensitive at baseline but can develop resistance upon continuous and prolonged treatment pressure with asparaginase. Leveraging outgrown Ptprk–Rspo3 organoids upon asparaginase treatment for RNA sequencing (Figure 4C), we could recapitulate our findings with a trend towards enrichment of RPL/RPS in downregulated transcripts (*p* = 0.1, Fisher’s exact test) when compared to vehicle-treated organoids (Figure 4D, Table S2).

### 2.5. Inhibition of Specific Ribosomal Proteins Promotes Cellular Fitness upon GSK3α Inhibition and Asparagine Starvation

**Figure 5:**
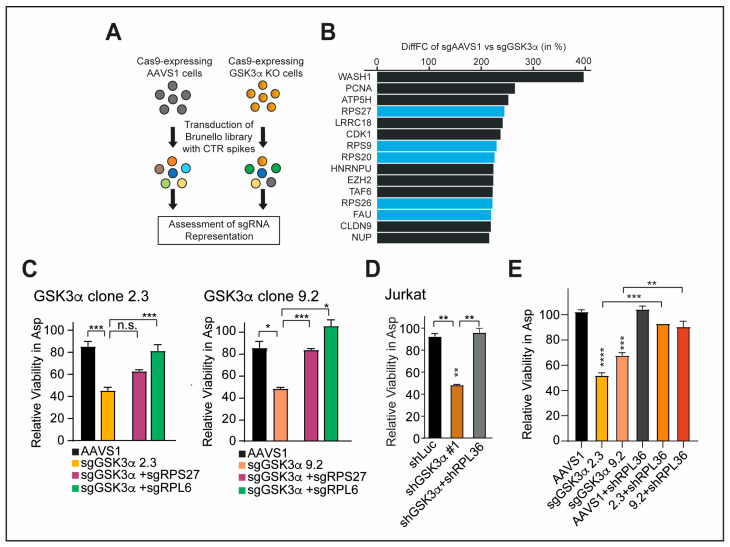
Inhibition of specific ribosomal proteins promotes cellular fitness upon GSK3α inhibition and asparagine starvation. (**A**) Schematic workflow of the CRISPR/Cas9 screen. (**B**) Top 15 genes that are differentially affected in amino-acid-deprived conditions between the two indicated cell lines from the experiment shown in (**A**). Ribosomal proteins are highlighted in blue. (**C**) GSK3α-KO single-cell clones were transduced with indicated sgRNAs and treated with vehicle or 100 U/L of asparaginase in biological duplicates. Relative viability was assessed after 6 days of treatment by counting viable cells. All cell counts were normalized to vehicle-treated cells. Note that asparaginase sensitization was not as striking because clones were chosen based on an intermediate response for the genome-wide CRISPR/Cas9 screen. Statistical significance was assessed using a one-way ANOVA with Dunnett’s adjustment for multiple comparisons. (**D**,**E**) Cells were transduced with indicated shRNAs and treated with vehicle or 100 U/L of asparaginase in biological duplicates. Relative viability was assessed after 6 days of treatment by counting viable cells. All cell counts were normalized to vehicle-treated cells. Note that asparaginase sensitization was not as striking because clones were chosen based on an intermediate response for the genome-wide CRISPR/Cas9 screen. Statistical significance was assessed using a one-way ANOVA with Dunnett’s adjustment for multiple comparisons. **** *p* ≤ 0.0001, *** *p* ≤ 0.001, ** *p* ≤ 0.01, * *p* < 0.05, and n.s. *p* ≥ 0.05.

To investigate the role of RPS/RPL in mediating cellular fitness in the context of GSK3α inhibition and asparaginase treatment, we generated GSK3α knockout (KO) as well as AAVS1 safe harbor control single-cell clones in Jurkat T-ALL cells (Figure S2B,C). Of note, we picked GSK3α KO single-cell clones with an intermediate asparaginase sensitization to allow screening for both sgRNA enrichment (resistance) and sgRNA dropout (exacerbated sensitization). Upon identification of suitable single-cell clones, we transduced these cells with a genome-wide sgRNA library (Brunello loss of function library), followed by treatment with vehicle or asparaginase (Figure S2D). Analysis of sgRNA representation revealed a significant enrichment of sgRNAs targeting ribosomal proteins when comparing GSK3α KO cells to AAVS1 cells in the presence of asparaginase treatment (*p* = 3.64 × 10^−7^, Fisher’s exact test) (Figure 5A,B, Tables S3 and S4).

For validation of selective RPS and RPL from both the transcriptomic approaches and the CRISPR/Cas9 screen, we focused on the top hits RPS27, RPL6, and RPL36, which have been shown to display anti-proliferative phenotypes, partially through a p53-dependent mechanism [47,49,50].

To validate that loss of RPL/RPS confers a survival advantage in GSK3α-inhibited cells, we lentivirally transduced sgRNAs targeting RPS27 and RPL6 in AAVS1 as well as two independent GSK3α KO single-cell clones (Figure S2B–D). Efficient gene silencing was confirmed with a qRT-PCR (Figure S2E). Indeed, the inhibition of RPL/RPS with sgRNAs was able to block GSK3α-inhibition mediated asparaginase sensitization (Figure 5C). In line, the knockdown of RPL36 in T-ALL as well as in CRC cells could block asparaginase cytotoxicity upon GSK3α inhibition and asparagine starvation (Figure 5D,E and Figure S2F).

These results underline that loss of distinct RPS/RPL can confer a survival advantage in the context of GSK3α inhibition and asparagine starvation. Of note, our described findings are in line with previously published data showing that positively selected sgRNAs target preferentially RPS/RPL that are known to be downregulated in cancer cells due to their anti-proliferative effect [47,48].

Taken together, our findings suggest the vital role of the previously unrecognized regulation of ribosomal proteins in bridging GSK3α activity and tolerance of asparagine starvation.

## 3. Discussion

Eukaryotic cells possess an array of machinery evolved to tolerate a plethora of environmental stressors including amino-acid-starved conditions. The delicate balance of protein metabolism and catabolism through the proteasome depends on sufficient amino acid availability [51]. In the case of some cancer cells, such as leukemia and colorectal cancer, this factor is detrimental in determining their survivability. Activation of the Wnt/STOP pathway halts GSK3α-dependent proteasomal degradation making the cells susceptible to amino acid deprivation as they heavily depend on these nutrients [6,7,8]. Accordingly, cell death in response to proteasome inhibition has been linked to a lethal amino acid shortage [52]. While these findings highlight the unique link between amino acid deprivation and cell death, the molecular components defining the link remain an important aspect to be investigated. Particularly since tolerance toward GSK3α inhibition and asparagine deprivation can still develop in these cancer cells.

Synthesis of components of the translational machinery represents a large part of the energetic costs of cellular life. Many feedback mechanisms have been discovered that link the production of different ribosomal components to maintain appropriate homeostasis [32,47]. Interestingly, the expression of distinct ribosomal proteins has been linked to either a tumorigenic or anti-proliferative phenotype [33,47,48]. The striking patterns of ribosomal protein expression across different cellular contexts thus highlight the diverse role of individual RPs. So far, at least three possible distinct mechanisms have been proposed to be involved including (i) global change in synthesis rate [53,54], (ii) modulation of translation rates of specific mRNAs with ribosomal proteins [55,56,57], and (iii) other extra-ribosomal functions of specific ribosomal proteins [32].

The studies shown here demonstrate that GSK3α-mediated asparaginase sensitization is independent of the GCN2-CHOP dependent axis, changes in cell cycle progression, and expression levels of ASNS. Of note, treatment with the elongation inhibitor homoharringtonine could also not rescue GSK3α-inhibited cells from asparaginase cytotoxicity, suggesting that driving protein translation with subsequent ATP depletion is not causing the described phenotype [25]. Strikingly, we found that the inhibition of GSK3α downregulates the expression of distinct RPS and RPL that are known to display anti-proliferative effects as part of extra-ribosomal functions. In line, the inhibition of these specific RPS and RPL could promote cellular fitness upon the inhibition of GSK3α and asparagine starvation.

It will be of considerable interest to investigate the molecular underpinnings of how GSK3α activity is interconnected with distinct RPS and RPL, and how these distinct RPs molecularly regulate GSK3α-mediated response to asparaginase. While the oncogenic effect upon the downregulation of specific RPS and RPL has been linked to a p53-dependent axis, previous studies also show that reliance on Wnt/STOP can depend on the p53 status of cancer cells [6]. Thus, it will be important to understand the role of p53 in the interconnection between GSK3α and specific ribosomal proteins.

Additionally, our studies indicate that driving protein translation with subsequent ATP depletion, which has been demonstrated by the forced expression of CHOP [25], is not causing cell death in GSK3α-inhibited cells. However, it remains unclear whether the downregulation of distinct RPS and RPL is regulating the cellular response solely through extra-ribosomal functions, or whether effects on protein synthesis are also of importance upon amino acid deprivation in a manner independent of the GCN2-CHOP axis.

Defects in ribosome biogenesis and function account for the pathogenesis of a variety of diseases called ribosomopathies, which are generally defined as diseases caused by mutations in the RPs [58,59,60,61]. It is of considerable interest that patients with ribosomopathies have an elevated risk of developing cancer throughout their life, despite the hypo-proliferative phenotypes associated with the early stage of the disease. The paradoxical transition from an early hypo-proliferative cellular response to a hyper-proliferative state is also best known as Dameshek’s riddle [62], linking defects between ribosome biogenesis and oncogenic transformation. Thus, a speculative but exciting possibility is that the activity of GSK3α plays a key role in Dameshek’s riddle.

Given the tremendous investment of cellular resources in the production of ribosomes, and the fact that a decrease in ribosome abundance protects cells against proteotoxic stress [63,64], a novel regulatory pathway—termed ribosomal assembly stress response (RASTR) [65]—has been recently demonstrated to play a crucial role in allowing the downregulation of RPs to save energy and re-establish cellular homeostasis upon cellular stress. It will be of future interest to investigate whether RASTR plays a role in mediating cellular fitness upon the knockdown of RPs in GSK3α-inhibited cancer cells, and if so, how exactly the downregulation of distinct RPS/RPL proteins regulates proteostasis capacity in these cells.

Collectively, our findings suggest that distinct RPS and RPL proteins play a crucial role in driving dependence toward the GSK3α-dependent proteasomal degradation machinery upon asparagine starvation in cancer cells.

## 4. Materials and Methods

### 4.1. Cell Lines, Cell Culture, and Organoids

293T cells, Jurkat, and HCT15 cells were obtained from ATCC (Manassas, VA, USA) DSMZ (Braunschweig, Germany) and cultured in DMEM or RPMI 1640 (Thermo Fisher Scientific, Karlsruhe, Germany) with 10% fetal bovine serum (FBS, Sigma-Aldrich, Saint Louis, MO, USA) and 1% penicillin/streptomycin (Thermo Fisher Scientific) at 37 °C and 5% CO_2_.

Organoids carrying *Ptprk–Rspo3* fusion were derived from transgenic mice and cultured as previously described [6]. Briefly, organoids were resuspended in Matrigel consisting of 25% advanced DMEM/F12 (Gibco, Karlsruhe, Germany) and 75% Matrigel (Corning, NY, USA). After polymerization, organoids were cultured in an organoid medium containing advanced DMEM/F-12 (Thermo Fisher Scientific), 1% penicillin/streptomycin, 2 mmol/L of l-Glutamine (Sigma-Aldrich, Darmstadt, Germany), 1 mmol/L of N-acetylcysteine (Sigma-Aldrich), and 10 mmol/L of HEPES (Sigma-Aldrich) supplemented with murine Noggin (50 ng/mL), murine EGF (R&D Systems, Wiesbaden, Germany, 50 ng/mL), and human RSPO1 (R&D Systems), as described previously [6]. All experiments involving asparaginase treatment were performed without growth factor supplementation.

Cell lines of early passages were exclusively used for these studies, and mycoplasma contamination was excluded using the MycoAlert Mycoplasma Detection Kit according to the manufacturer’s instructions (Lonza, Rockland, WA, USA; most recently in November 2022).

### 4.2. Lentiviral Transduction

Lentiviral vectors were generated using co-transfecting pLKO.1 plasmid of interest together with packaging vectors psPAX2 (a gift from Didier Trono; Addgene plasmid # 12260) and VSV.G (a gift from Tannishtha Reya; Addgene plasmid # 14888) using OptiMEM (Invitrogen, Karlsruhe, Germany) and polyethyleneimine (Sigma-Aldrich), as previously described [7]. For a concentrated virus, the virus-containing medium was ultracentrifuged at 24.000 rpm for 2 h at 4 °C (Beckman Coulter, Krefeld, Germany), and the obtained pellet was resuspended in RPMI.

Lentiviral infections with the unconcentrated virus were performed with spinoculating cell lines with virus-containing media (1500 g × 90 min) in the presence of 8 μg/mL of polybrene (Merck Millipore, Darmstadt, Germany). Lentiviral infections with the concentrated virus were performed without spinoculation by directly adding the virus to the cultured cells.

Selection with antibiotics was started 24 h after infection with neomycin (700 μg/mL for a minimum of 5 days; Thermo Fisher Scientific), puromycin (1 μg/mL for a minimum of 48 h; Thermo Fisher Scientific), or blasticidin (15 µg/mL for a minimum of 5 days; Invivogen, Toulouse, France).

### 4.3. Short Hairpin RNA (shRNA), Single-Guide RNA (sgRNA), and Expression Plasmids

The following lentiviral shRNA vectors in pLKO.1 with puromycin were generated by the RNAi Consortium library and obtained from Sigma-Aldrich as bacterial stocks. Alternatively, oligos were purchased from Eurofins or IDT and cloned in pLKO.1 with blasticidin (a gift from Keith Mostov, Addgene plasmid #26655). The shRNA sequences are as follows: shLuciferase (TRCN0000072243), shCHOP #1 (TRCN0000007263), shCHOP #2 (TRCN0000007264), shGCN2 #1/shEIF2AK4 #1 (TRCN0000078649), shGCN2 #3/shEIF2AK4 #3 (TRCN0000078652), shGSK3α #1 (TRCN0000010340), shGSK3α #6 (TRCN0000038681), and shRPL36 (TRCN0000117674).

For CRISPR/Cas9, *GSK3α* sgRNAs were designed using the CRISPick tool by Broad Institute [66,67]. *AAVS1* sgRNAs were based on a previous publication [68]. Oligos were purchased from Eurofins or IDT and cloned in LentiGuide-Neo backbone (a gift from Caroline Goujon, Addgene plasmid #139449).

GFP sequence was based on *A*. *victoria* GFP mRNA (M62653.1). DNA constructs were designed with attB sites for gateway cloning and subsequently cloned into the pLX304 destination vector (a gift from David Root; Addgene plasmid #25890). For Gateway cloning of codon-optimized GSK3α, wild-type constructs were designed with attB sites and subsequently cloned into the pLX304 destination vector (a gift from David Root; Addgene plasmid #25890). The amino acid sequence of wild-type GSK3α is based on UniProt identifier P49840 (GSK3α). Note that GSK3α cDNA expression constructs escape targeting via the GSK3α shRNA, which targets the 3′UTR of endogenous GSK3α.

A hyperactive open-gate mutant of the human proteasomal subunit PSMA4, termed ∆N-PSMA4, was designed by deleting the cDNA sequences encoding amino acids 2 to 10 (SRRYDSRTT) of PSMA4 isoform NP_002780.1 (encoded by the transcript NM_002789.6), based on the data of Choi and colleagues [46]. This ∆N-PSMA4 coding sequence was synthesized via gene synthesis and cloned into the pLX304 lentiviral expression vector in-frame with the C-terminal V5 tag provided by this vector, by GeneCopoeia.

### 4.4. Assessment of Chemotherapy Response

Leukemia cells were seeded at 25.000 cells per well in 100 µL of complete growth medium in 96-well plates and incubated with indicated chemotherapeutic agent or vehicle. T-ALL cells were split every 48 h. Briefly, 20 µL of cells were mixed with 80 µL of fresh culture medium, supplemented with vehicle or chemotherapeutic drugs at the indicated doses.

For the treatment of HCT15 cells, 250.000 cells were seeded in 2.5 mL of culture medium supplemented with the indicated drugs in a 12-well or 6-well plate format, respectively. Cell viability was assessed by counting viable cells based on trypan blue vital dye staining (Invitrogen), according to the manufacturer’s instructions.

For the treatment of organoids, Matrigel and basal organoid medium without growth factors were supplemented with vehicle or 100 U/L of asparaginase and split every 48 h.

All asparaginase experiments were performed using pegaspargase (Oncaspar, Shire Pharmaceuticals, Lexington, MA, USA), an FDA-approved PEGylated form of E. coli asparaginase. Homoharringtonine (SML1091) was obtained from Sigma-Aldrich. All drugs and reagents were used at the indicated concentrations.

### 4.5. Quantitative Reverse Transcriptase PCR (qRT-PCR)

RNA was isolated using the RNeasy kit (Qiagen, Hilden, Germany), and cDNA was made using the RevertAid First-Strand cDNA synthesis kit (Thermo Fisher Scientific). qRT-PCR was performed using the iTaq Universal SYBR^®^ Green Supermix (Biorad) and QuantStudio 1 Real-Time PCR system (Applied Biosystems, Hamburg, Germany). The primers used are described in Table S5.

### 4.6. Western Blot Analysis

Cells were lysed in RIPA buffer (Merck Millipore) supplemented with cOmplete protease inhibitor (Roche, Mannheim, Germany) and PhosSTOP phosphatase inhibitor (Roche). Laemmli sample buffer (Bio-Rad, Feldkirchen, Germany) and β-mercaptoethanol (Sigma-Aldrich) was mixed with protein lysate before being run on a 4% to 12% bis-tris polyacrylamide gel (Bio-Rad). Blots were transferred to polyvinylidene difluoride (PVDF) membrane (Carl Roth, Karlsruhe, Germany) and blocked with 5% BSA (AppliChem, Darmstadt, Germany) or 5% sure block (LubioScience, Karlsruhe, Germany) in PBS with 0.1% Tween, and they were probed with the following antibodies: K48-linked ubiquitin (1:1000, Abcam, Berlin, Germany, #ab140601) and GAPDH (1:1000, Cell Signaling, Leiden, Netherlands #2118). Detection of horseradish peroxidase-linked secondary antibodies (mouse and rabbit) with horseradish peroxidase substrate (Santa Cruz, Heidelberg, Germany, #sc-516102 and #sc-2357) was visualized using Amersham Imager 800 (Cytiva, Freiburg, Germany).

### 4.7. Cell Cycle Analysis

Cells were harvested, and the supernatant was removed. Cell pellets were fixed with 4% paraformaldehyde for 10 min at room temperature and subsequently washed with PBS. Staining was performed with FxCycle™ PI/RNase Staining Solution (Thermo Fisher Scientific) according to the manufacturer’s protocol. Samples were analyzed using the BD FACS Canto II instrument (BD Biosciences, Heidelberg, Germany).

### 4.8. Caspase 3/7 Activity and BH3 Profiling

Caspase 3/7 activity was assessed using the Caspase Glo 3/7 Assay (Promega, Mannheim, Germany) according to the manufacturer’s instructions.

BH3 profiling was performed as previously described [69]. Briefly, 100,000 cells were incubated with 1 µM of BIM peptide (Eurogentec, Seraing, Belgium) in MEB buffer (150 mM mannitol, 50 mM KCl, 0.02 mM EDTA, 0.02 mM EGTA, 5 mM succinate, 0.1% BSA, and 10 mM HEPES-KOH; final pH, 7.5) containing 0.002% (wt/vol) of digitonin (Sigma-Aldrich) for 45 min. Cells were then fixed in 4% paraformaldehyde (Alfa Aesar, Kandel, Germany). Cytochrome c was stained using 1:500 anti-cytochrome c antibody overnight at 4 °C. The following day, cells were washed and stained with Alexa Fluor 555 (Thermo Fisher Scientific) for 1 h, followed by analysis using flow cytometry with the BD FACS Canto II instrument (BD Biosciences).

### 4.9. Timeseries RNA Sequencing and Bioinformatic Analysis

Cells were treated with indicated drugs, and total RNA was isolated at indicated time points using the RNeasy kit (Qiagen) according to the manufacturer’s protocols. RNA sequencing was performed at Eurofins genomics (Genome Sequencer Illumina NovaSeq 6000, S4 PE150 XP).

Initial quality control of transcriptomics data was performed using FastQC version 0.11.9 (https://www.bioinformatics.babraham.ac.uk/projects/fastqc/, accessed on 27 April 2023). After the assessment, the “filter by tile” function of BBMap [70] was used to discard low-quality reads, before trimming with trimmomatic version 0.39 [71]. Orphaned reads were merged to their respective paired fastq files using the cat command of Ubuntu 22.04.1 LTS.

The preprocessed reads were subsequently mapped using kallisto version 0.46.1 [72] with a transcriptome index created using Homo_sapiens.GRCh38.cdna.all.fa.gz (https://ftp.ensembl.org/pub/current_fasta/homo_sapiens/cdna/; last modified 13 December 2022 11:30) [73]. For further analysis, kallisto-estimated counts of protein-coding transcripts that are associated with a specific HGNC accession (https://www.genenames.org/, accessed on 27 April 2023) were used. Transcripts were categorized as protein-coding or non-protein-coding using Ensembl [73] gene biotype information. Transcripts with labels “IG_C_gene”, “IG_D_gene”, “IG_V_gene”, “IG_J_gene”, “protein_coding”, “TR_D_gene”, “TR_J_gene”, or “TR_V_gene” were considered as protein-coding. Counts were discarded for transcripts with a count < 10 across all analyzed samples. The remaining transcripts were normalized to obtain transcript per million (TPM) values. TPMs of transcripts associated with the same HGNC accession were added up to calculate gene level expression. On the gene level, a zTPM cut-off was used to exclude lowly expressed genes and their transcripts, as previously described [74].

### 4.10. Organoids RNA Sequencing and Bioinformatic Analysis

Cells were treated with indicated drugs, and total RNA was isolated at the indicated time point using the RNeasy kit (Qiagen) according to the manufacturer’s protocols. RNA sequencing was performed at Eurofins genomics (Genome Sequencer Illumina NovaSeq 6000, S4 PE150 XP).

Initial quality control was performed using FastQC version 0.11.9 (https://www.bioinformatics.babraham.ac.uk/projects/fastqc/, accessed on 27 April 2023 ). Followed by discarding low-quality reads using the “filter by tile” function of BBMap [70] and trimming with trimmomatic version 0.39 [71]. The pre-processed reads were subsequently mapped using kallisto version 0.46.1, with a prebuilt murine transcriptome index (https://github.com/pachterlab/kallisto-transcriptome-indices/releases, accessed on 27 April 2023) [72]. For further analysis, kallisto-estimated counts of protein-coding transcripts were used. Transcripts were categorized as protein-coding or non-protein-coding using Ensembl [73] gene biotype information. Transcripts with labels “IG_C_gene”, “IG_D_gene”, “IG_V_gene”, “IG_J_gene”, “protein_coding”, “TR_D_gene”, “TR_J_gene”, or “TR_V_gene” were considered as protein-coding.

### 4.11. CRISPR/Cas9 Loss of Function Screen

Jurkat T-ALL cells were transduced with lentiCas9-blast (a gift from Feng Zhang; Addgene plasmid #52962), selected with blasticidin, and Cas9 activity was confirmed using a self-excising GFP construct, pXPR_011 (a gift from John Doench and David Root; Addgene plasmid #59702). Upon confirmation, Jurkat-Cas9 cells were transduced with sgRNAs targeting *AAVS1* [68] or *GSK3α* containing neomycin selection markers (a gift from Caroline Goujon; Addgene plasmid #139449). Cells were then subjected to a limiting dilution strategy to obtain single-cell clones of the desired phenotype. Subsequently, GSK3α-knockout clones (KO) were confirmed based on GSK3α mRNA levels, GSK3α protein expression levels, and sensitization to asparaginase.

The genome-wide CRISPR/Cas9 screen was performed by utilizing the human Brunello CRISPR knockout pooled library (a gift from David Root and John Doench; Addgene #73178). The library was “spiked” with control sgRNAs including *ASNS* as a control to reduce false positives and false negatives based on previous publications [5]. The Brunello library with “spikes” was transduced in biological triplicates into AAVS1 and GSK3α-KO cells at a multiplicity of infection of 0.3. Cells were then selected with puromycin (1 µg/mL) beginning 24 h post transduction, which was continued for 8 days. Cells were split every other day, and the minimum number of cells kpt at each split was maintained at 0.4 × 10^6^ per mL to minimize loss of guide RNA coverage. Cells were treated with 100 U/L of asparaginase beginning on day 10 and were harvested after 4 days of treatment. Genomic DNA was extracted using the Blood & Cell Culture DNA Maxi Kit (Qiagen) according to the manufacturer’s protocols. Samples were PCR-amplified for the gRNA cassette, followed by attaching Illumina-suitable primers. Samples were sequenced using Next-Generation Sequencing performed at Eurofins genomics (Genome Sequencer Illumina MiSeq Personal Sequencer, 300 bp paired-end).

Demultiplexed paired-end reads of the screen sequencing data were provided by Eurofins Genomics. Initial quality control was performed using FastQC version 0.11.9 (https://www.bioinformatics.babraham.ac.uk/projects/fastqc/, accessed on 27 April 2023). For further analysis, only reads generated from the forward primer were considered, since the sgRNA target sequence was not contained in reads generated using the reverse sequencing primers. Reads were then trimmed to a length of 75 bp using trimmomatic version 0.39 [71]. The count function of MAGeCK version 0.5.9.5 was used [75], with a modified Brunello library and the additional arguments –unmapped-to-file, –sgrna-len 20, and –trim-5 34 to obtain normalized sgRNA target sequence counts (Table S4). The modified Brunello library contains additional sgRNA target sequences for added controls (so-called “spikes”). For statistical analysis, the MAGeCK test function was used with the previously generated normalized counts. The MAGeCK “gene.summary” results contain log fold changes describing the difference in sgRNA target abundance between treatment and vehicle conditions. To analyze differences in the effect of asparaginase treatment on-screen results between sgAAVS1 and sgGSK3α a differential fold change (diffFC) in percent was calculated using Formula (1). Here, aFC describes the absolute fold change between normalized counts of a given gene in a vehicle or asparaginase-treated sgAAVS1 cells, with gFC describing the corresponding value in sgGSK3α cells.

diffFC = (gFC* 100)/aFC(1)

### 4.12. Quantification and Statistical Analysis

A two-tailed Welch unequal variances t-test was used for two-group comparisons of continuous measures. For 3-group comparisons, a one-way analysis of variance model (ANOVA) was performed, and a Dunnett adjustment for multiple comparisons was used. Data shown as bar graphs represent the mean and standard error of the mean (s.e.m) of a minimum of 2 biological replicates. All *p*-values reported are two-sided and considered significant if <0.05.

## Data Availability

The raw sequencing data of long-term asparaginase treatment, as well as of asparaginase-treated CRC organoids, can be found in the NCBI GEO database with the accession numbers GSE234800, GSE234801, and GSE234803, respectively. Processed data of the CRISPR/Cas9 screen shown in this study can be found in supplementary Tables S3 and S4. Raw data from the screen are available upon reasonable request from the corresponding author.

## Supplementary Materials

The following supporting information can be downloaded at https://www.mdpi.com/article/10.3390/ijms241713260/s1.
