# Supplementary material for: GSK3α Regulates Temporally Dynamic Changes in Ribosomal Proteins upon Amino Acid Starvation in Cancer Cells
Source: Int J Mol Sci. 2023 Aug 26;24(17):13260. doi: 10.3390/ijms241713260 (PMC10488213; doi:10.3390/ijms241713260)
Supplement: Supplementary file 1 [file ijms-24-13260-s001.zip › Supplementary Files/full western blot images.pdf]

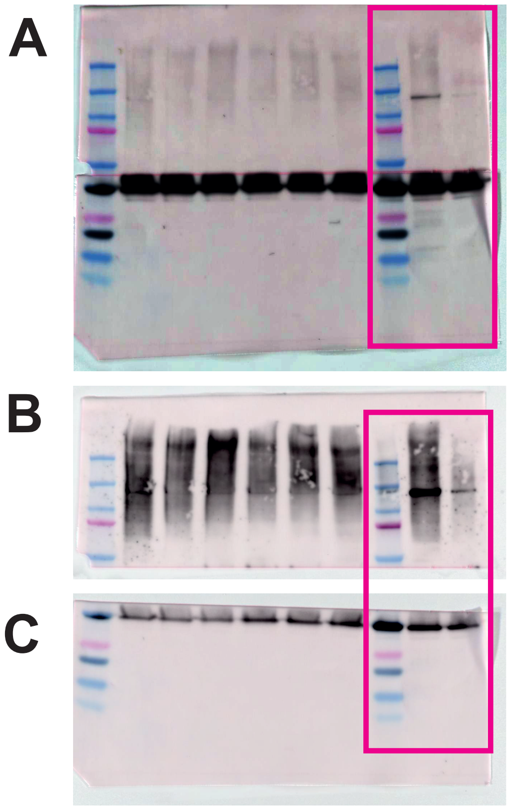

A. Full Western blot image from Figure 1C
B. Top portion of blot stained for K48-linked Ubiquitin
C. Bottom portion of blot stained for GAPDH
Blots were imaged separately for individual exposure times
Cropped blot in figure is of lane 9 and 10 as marked.

Loading order:
1. Protein Ladder
2. Jurkat shLuc Vehicle
3. Jurkat shPRUNE #2 Vehicle
4. Jurkat shPRUNE #5 Vehicle
5. Jurkat shLuc Asparaginase
6. Jurkat shPRUNE #2 Asparaginase
7. Jurkat shPRUNE #5 Asparaginase
8. Protein Ladder
9. Jurkat shLuc
10. Jurkat shGSK3α

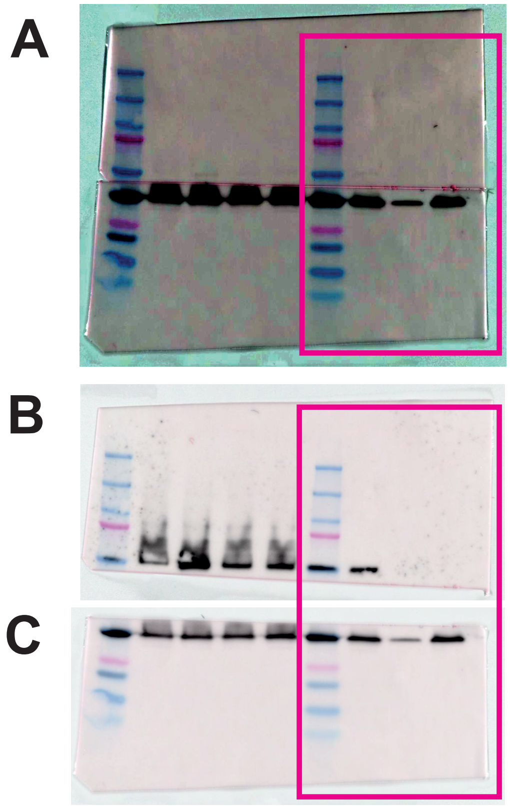

A. Full Western blot image from Supplementary Figure 2C
B. Top portion of blot stained for GSK3α
C. Bottom portion of blot stained for GAPDH
Blots were imaged separately for individual exposure times
Cropped blot in figure is of lane 7, 8 and 9 as marked.

Loading order:
1. Protein Ladder
2. Jurkat shLuc
3. Jurkat shPRUNE #2
4. Jurkat shPRUNE #5
5. Jurkat
6. Protein Ladder
7. Jurkat AAVS1
8. Jurkat sgGSK3α #2.3
9. Jurkat sgGSK3α #9.2

A

B

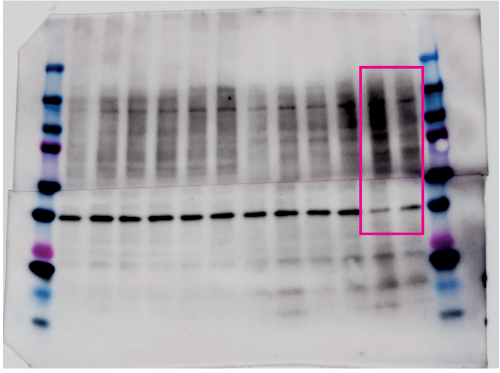

Full Western blot image from Supplementary Figure 1E

A. Top portion of blot stained for K48-linked Ubiquitin
B. Bottom portion of blot stained for GAPDH

Cropped blot in figure is of lane 12 and 13 as marked

Loading order:

1. Protein Ladder
2. CEM
3. CEM clone 1
4. CEM clone 2
5. NALM-16
6. NALM-16 clone 1
7. NALM-16 clone 2
8. KOPTK1
9. KOPTK1 clone 1
10. KOPTK1 clone 2
11. Jurkat
12. HCT-15 shLuc Asp
13. HCT-15 shGSK3a Asp
14. Protein Ladder
