# Supplementary material for: GSK3α Regulates Temporally Dynamic Changes in Ribosomal Proteins upon Amino Acid Starvation in Cancer Cells
Source: Int J Mol Sci. 2023 Aug 26;24(17):13260. doi: 10.3390/ijms241713260 (PMC10488213; doi:10.3390/ijms241713260)
Supplement: Supplementary file 1 [file ijms-24-13260-s001.zip › Supplementary Files/Supplementary Materials 2023.08.13.docx]

Supplementary materials

GSK3α Regulates Temporally Dynamic Changes in Ribosomal Proteins Upon Amino Acid Starvation in Cancer Cells

Lorent Loxha ^1, †^, Nurul Khalida Ibrahim ^1, †^, Anna Sophie Stasche ^1^, Büsra Cinar ^1^, Tim Dolgner ^1^, Julia Niessen ^1^, Sabine Schreek ^1^, Beate Fehlhaber ^1^, Michael Forster ^2^, Martin Stanulla ^1^, and Laura Hinze ^1,^ *

^1^ Department of Pediatric Hematology and Oncology, Hannover Medical School, Hannover 30625, Germany; Loxha.Lorent@mh-hannover.de (L.L); Ibrahim.Nurul@mh-hannover.de (N.K.I.); Stasche.Anna@mh-hannover.de (A.S.); Cinar.Buesra@mh-hannover.de (B.C.); [Dolgner.Tim@mh-hannover.de](mailto:Dolgner.Tim@mh-hannover.de) (T.D.); Niessen.Julia@mh-hannover.de (J.N.); Schreek.Sabine@mh-hannover.de (S.S.); Fehlhaber.Beate@mh-hannover.de (B.F.); Stanulla.Martin@mh-hannover.de (M.S.); Hinze.Laura@mh-hannover.de (L.H.).

^2^ Institute of Clinical Molecular Biology, Kiel University, Kiel 24105, Germany; m.forster@ikmb.uni-kiel.de (M.F.)

***** Correspondence: hinze.laura@mh-hannover.de

† These authors contributed equally to this work.

**Supplementary Materials**

**Supplementary Figures**


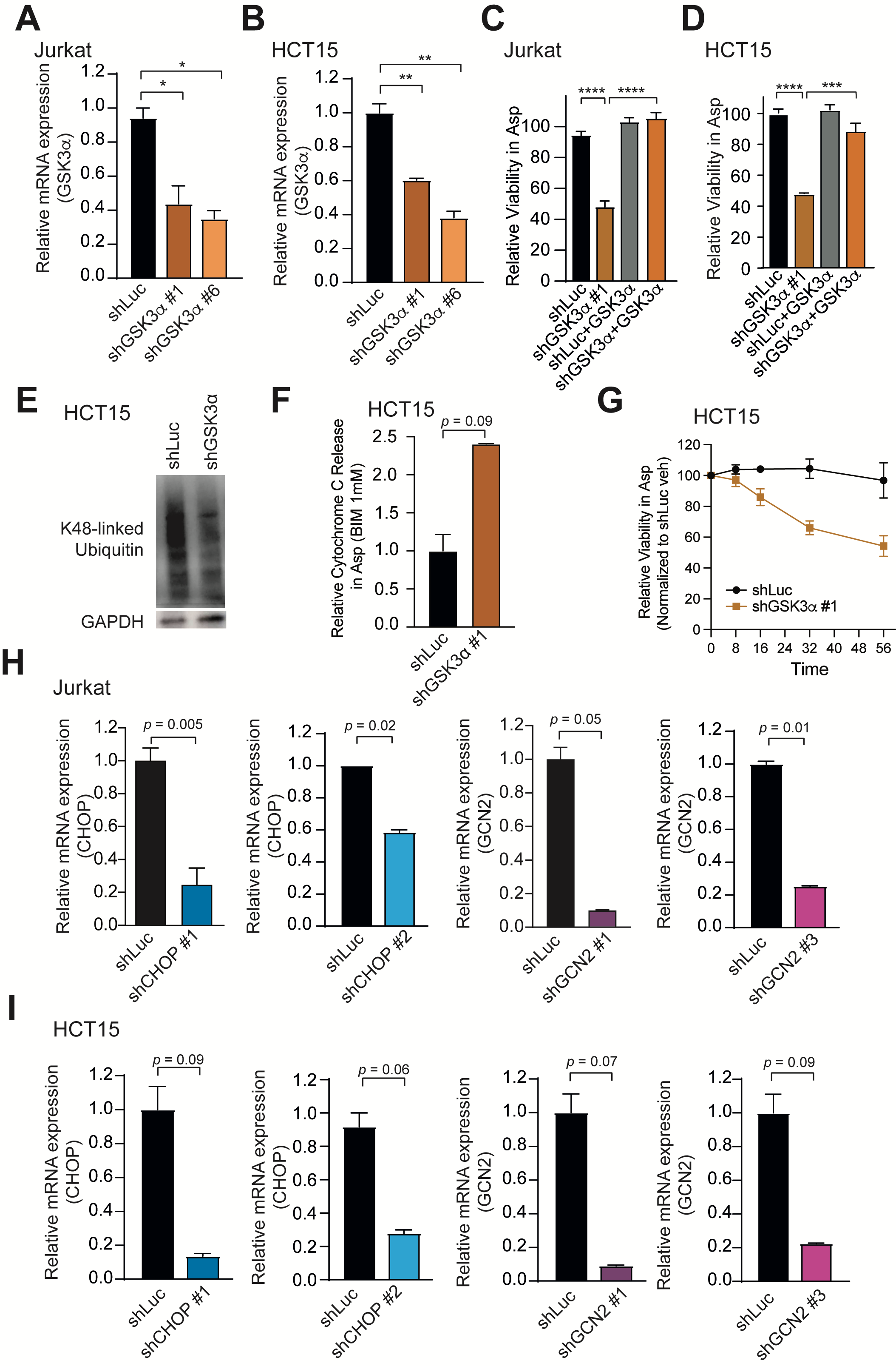


**Figure S1.** Supplementary data related to Figures 1 and 2. **A-B)** Jurkat cells and HCT15 cells were transduced with indicated shRNAs, and knockdown efficiency was assessed by qRT-PCR analysis in biological duplicates. Statistical significance was assessed using a one-way ANOVA with Dunnett’s adjustment for multiple comparisons. **(C-D)** Indicated cells were transduced with indicated constructs and treated with vehicle or 100 U/L asparaginase in biological triplicates. Relative viability was assessed after 8 days of treatment by counting viable cells. Cell counts were normalized to shLuc, vehicle treated cells. Statistical significance was assessed using a one-way ANOVA with Dunnett’s adjustment for multiple comparisons. (**E**) HCT15 cells were transduced with indicated shRNAs. After knockdown validation, protein levels of K48-linked ubiquitin and GAPDH were assessed by Western blot analysis upon asparaginase treatment (100 U/L). (**F**) Cells were transduced with indicated shRNAs, and treated with 100 U/L asparaginase for 48 hours, and cytochrome C release was assessed in biological duplicates. Statistical significance was assessed using a two-sided Student’s t-test with Welch adjustment (**G**) HCT15 cells were transduced with indicated shRNAs, and treated with vehicle or 100 U/L asparaginase in biological triplicates. Relative viability was assessed at indicated time points by counting viable cells. All cell counts were normalized to shLuc-transduced, vehicle-treated cells. Note that the sensitization phenotype is not as striking as in Figure 1F as colorectal cancer cells require prolonged asparagine starvation upon inhibition of GSK3α (Hinze et al., Cancer Discovery, 2020). **H-I)** Jurkat cells and HCT15 cells were transduced with indicated shRNAs, and knockdown efficiency was assessed by qRT-PCR analysis in biological duplicates. Statistical significance was assessed using a two-sided Student’s t-test with Welch adjustment. All error bars represent SEM. **** p ≤ 0.0001, *** p ≤ 0.001, ** p ≤ 0.01; * p < 0.05.


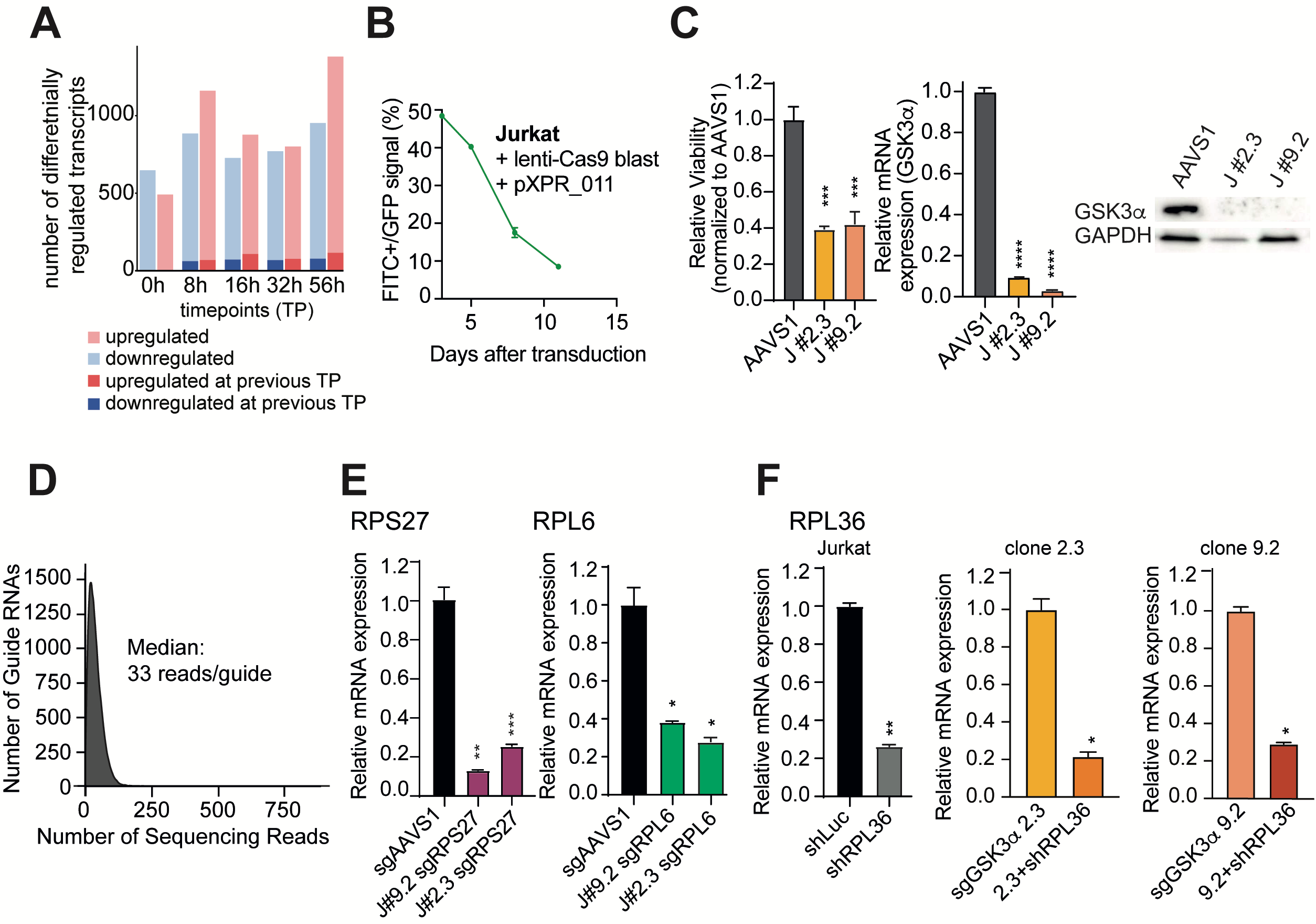


**Figure S2.** Supplementary data related to Figures 4 and 5. **A)** Bar plot showing differentially up- or down-regulated transcripts that are unique to the given time point, or regulated in the same direction at the previous time point. **B)** Jurkat T-ALL cells were transduced with Cas9 and pXPR_011 virus and the GFP signal was assessed 2-, 4-, and 9-days post-selection in biological triplicates. **C)** Functional validation of GSK3α-KO clones 2.3 and 9.2 as assessed by relative viability in 100 U/L asparaginase treatment after 6 days, qRT-PCR knockdown, and protein levels by Western blot. Statistical significance was assessed by a one-way ANOVA with Dunnett’s adjustment for multiple comparisons. **D)** Brunello library sgRNA representation assessed using next-generation sequencing. **E)** GSK3α-KO clones were transduced with indicated sgRNAs, and knockdown efficiency was assessed by qRT-PCR analysis in biological duplicates. Statistical significance was assessed by a one-way ANOVA with Dunnett’s adjustment for multiple comparisons. **F)** Jurkat cells, or indicated clones were transduced with shRPL36 and knockdown efficiency was assessed by qRT-PCR analysis in biological duplicates. Statistical significance was assessed using a two-sided Student’s t-test with Welch adjustment. All error bars represent SEM. **** p ≤ 0.0001; *** p ≤ 0.001; ** p ≤ 0.01; * p < 0.05.

**Supplementary Tables (Tables in Excel file)**

**Table S1.** Transcriptomics analysis from the time-series RNA sequencing

**Table S2**. List of downregulated transcripts from RNA sequencing of RSPO3 fusion organoids

**Table S3.** List of differentially expressed genes from the CRISPR/Cas9 genome-wide screen with a differential fold-change threshold of above 200.

**Table S4.** Normalized counts from Table S3

**Table S5.** Primers and oligos
