# Supplementary figures and images for: GSK3α Regulates Temporally Dynamic Changes in Ribosomal Proteins upon Amino Acid Starvation in Cancer Cells
Source: Int J Mol Sci. 2023 Aug 26;24(17):13260. doi: 10.3390/ijms241713260 (PMC10488213; doi:10.3390/ijms241713260)

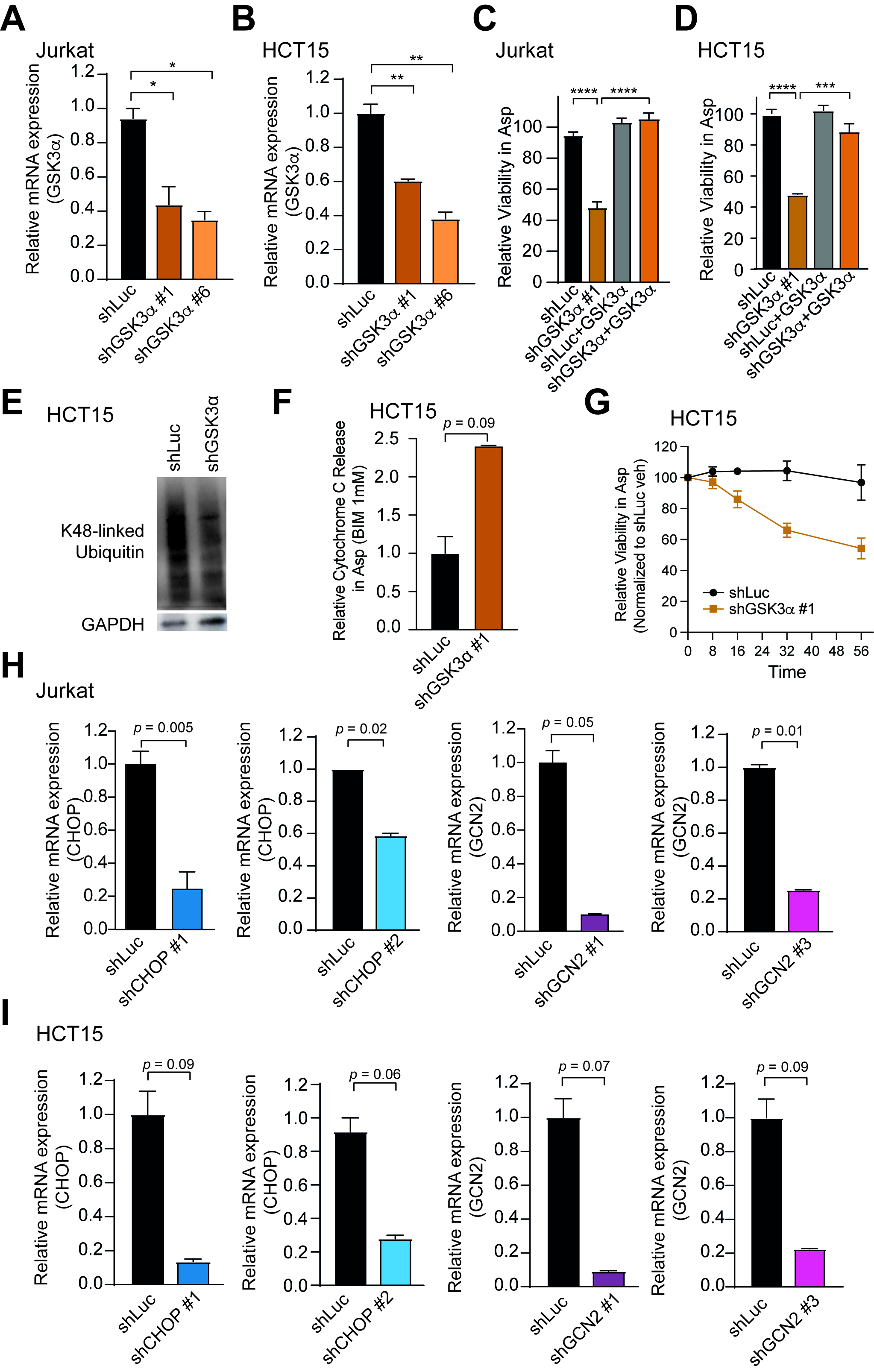

Supplement: Supplementary file 1 [file ijms-24-13260-s001.zip › Supplementary Files/Supplementary Figure 1 2023.08.14.tif]

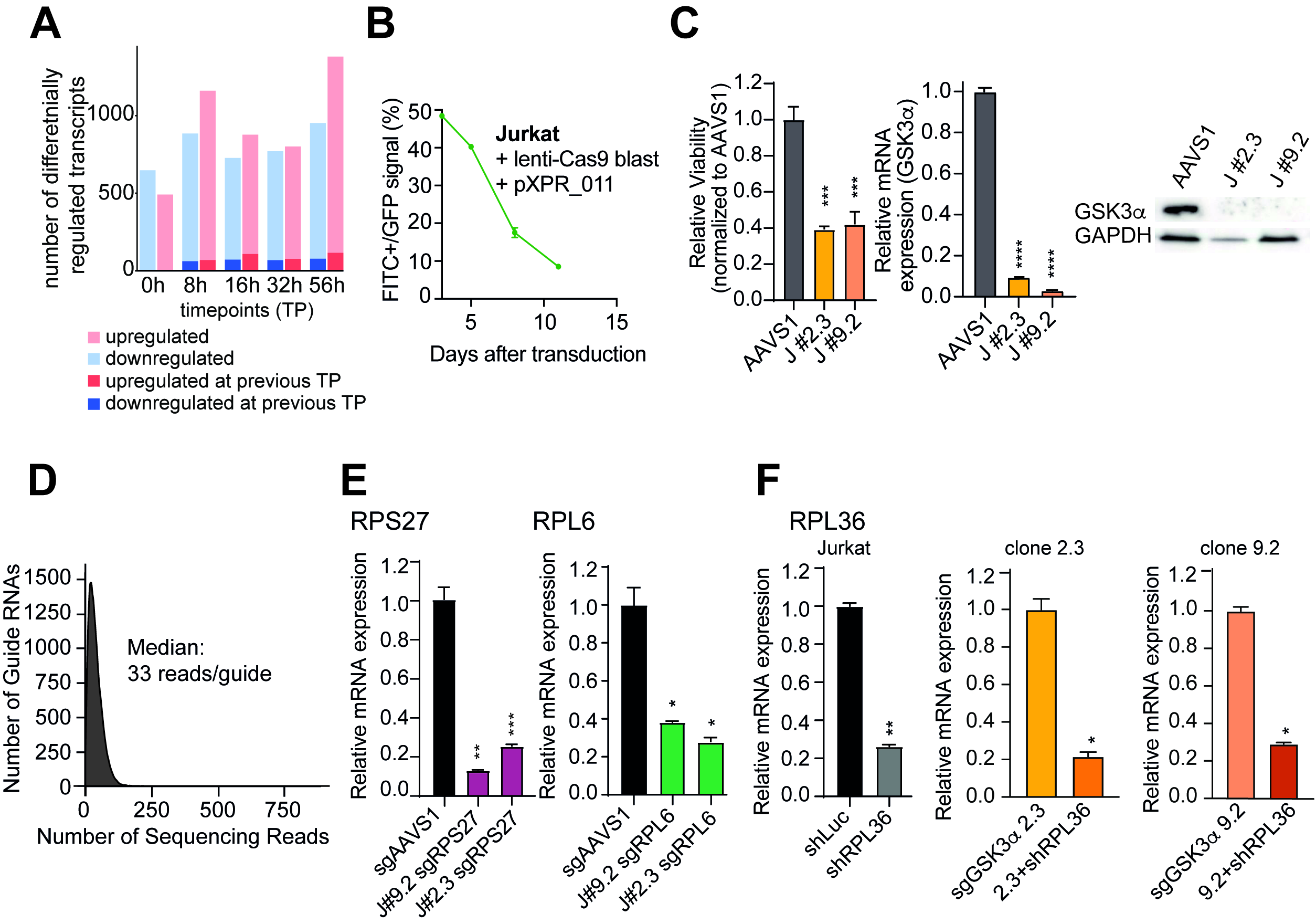

Supplement: Supplementary file 1 [file ijms-24-13260-s001.zip › Supplementary Files/Supplementary Figure 2 2023.08.14.tif]
